# 1,1′-(Butane-1,4-di­yl)bis­[2-(pyridin-2-yl)-1*H*-benzimidazole]

**DOI:** 10.1107/S1600536812015899

**Published:** 2012-04-18

**Authors:** Shao-Chuan Zhou, Hong-Zhen Xie

**Affiliations:** a State Key Lab. Base of Novel Functional Materials and Preparation Science, Faculty of Materials Science and Chemical Engineering, Ningbo University, Ningbo, Zhejiang, 315211, People’s Republic of China

## Abstract

The complete mol­ecule of the title compound, C_28_H_24_N_6_, is generated by inversion symmetry with the inversion centre located at the mid-point of the central C–C bond of the butanediyl unit. The benzimidazole and pyridine rings are almost coplanar, the dihedral angle between their mean planes being 6.86 (11)°.

## Related literature

For the synthesis, see: Liu *et al.* (2010 ▶). For background to this study, see: Barnett & Champness (2003 ▶); Tong *et al.* (2009 ▶).
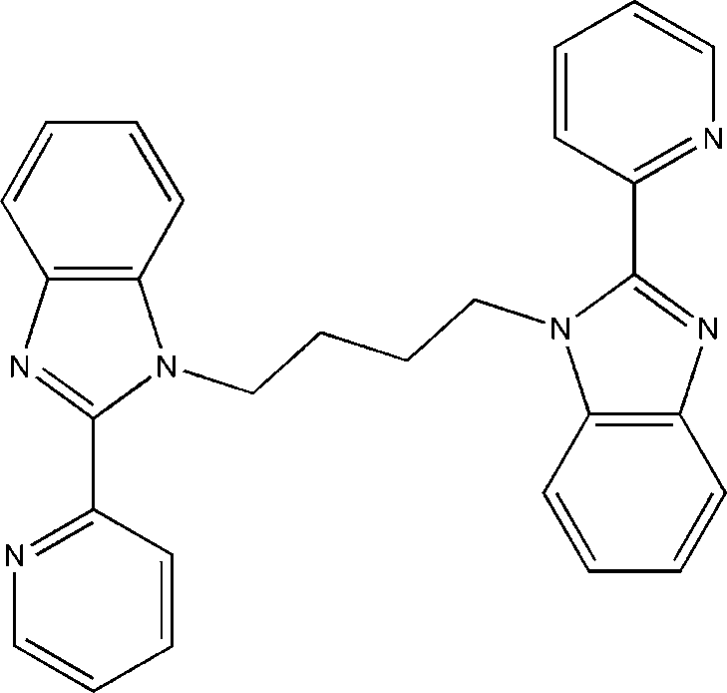


## Experimental

### 

#### Crystal data

C_28_H_24_N_6_
*M* *_r_* = 444.53
Monoclinic, 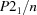

*a* = 6.5617 (7) Å
*b* = 13.9716 (13) Å
*c* = 12.3351 (8) Å
β = 96.466 (7)°
*V* = 1123.66 (17) Å^3^
*Z* = 2
Mo *K*α radiation
μ = 0.08 mm^−1^
*T* = 298 K
0.42 × 0.18 × 0.15 mm

#### Data collection

Rigaku R-AXIS RAPID diffractometer
Absorption correction: multi-scan (*ABSCOR*; Higashi, 1995 ▶) *T*_min_ = 0.695, *T*_max_ = 0.856
6268 measured reflections
2684 independent reflections
1314 reflections with *I* > 2σ(*I*)
*R*_int_ = 0.055

#### Refinement

*R*[*F* ^2^ > 2σ(*F* ^2^)] = 0.064
*wR*(*F* ^2^) = 0.146
*S* = 1.02
2684 reflections
162 parameters
H atoms treated by a mixture of independent and constrained refinement
Δρ_max_ = 0.34 e Å^−3^
Δρ_min_ = −0.20 e Å^−3^

### 

Data collection: *RAPID-AUTO* (Rigaku, 1998 ▶); cell refinement: *RAPID-AUTO*; data reduction: *CrystalStructure* (Rigaku/MSC, 2002 ▶); program(s) used to solve structure: *SHELXS97* (Sheldrick, 2008 ▶); program(s) used to refine structure: *SHELXL97* (Sheldrick, 2008 ▶); molecular graphics: *ORTEPII* (Johnson, 1976 ▶); software used to prepare material for publication: *SHELXL97*.

## Supplementary Material

Crystal structure: contains datablock(s) global, I. DOI: 10.1107/S1600536812015899/ff2063sup1.cif

Structure factors: contains datablock(s) I. DOI: 10.1107/S1600536812015899/ff2063Isup2.hkl

Supplementary material file. DOI: 10.1107/S1600536812015899/ff2063Isup3.cml

Additional supplementary materials:  crystallographic information; 3D view; checkCIF report

## Figures and Tables

**Fig. 1.**
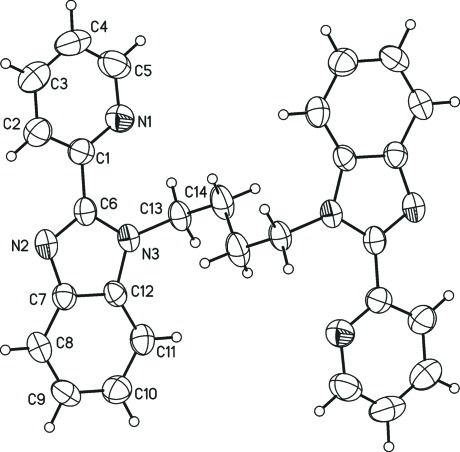
ORTEP view of complex molecule of (I). Displacement ellipsoids are drawn at the 45% probability level. H atoms were omitted for clarity.

## supplementary crystallographic information

### Comment

The long spacer ligands, particularly the flexible N-bridging donors have been investigated as auxiliary ligands for the construction of novel MOFs [Liu *et al.*, 2010; Barnett *et al.*, 2003; Tong *et al.*, 2009]. As part of our ongoing studies, the title compound was synthesized and characterized by X-ray diffraction.

The complete molecule of the title compound, C_28_H_24_N_6_, is generated by crystallographic inversion symmetry and the central C—C bond of the butanediyl unit is bisected by the inversion symmetry. The dihedral angle between the benzimidazole ring system and the pyridine ring is 6.86 (11)°, which indicates that they are almost coplanar.

### Experimental

According to the literature [Liu *et al.*, 2010], 2-(2-pyridyl)benzimidazole (7.80 g) and NaOH (1.68 g) in DMSO (20 ml) were stirred at 60°C for 0.5 h, and then 1,4-dibromobutane (4.32 g) was added. The mixture was stirred at 60°C for 12 h, and then poured into 400 ml of ice water after being cooled to room temperature. The yellow solid was obtained and isolated by filtration after drying in air. The above products were recrystallized in methanol and yellow crystals of the title compounds were obtained.

### Refinement

The H atoms bonded to C except for C14 were placed at calculated positions and refined in riding mode with *U*_iso_(H)=1.2Ueq(C). The H atoms of C14 were located at difference Fourier maps and refined freely.

### Crystal data

**Table:**

C_28_H_24_N_6_	*F*(000) = 468
*M**_r_* = 444.53	*D*_x_ = 1.314 Mg m^−^^3^
Monoclinic, *P*2_1_/*n*	Mo *K*α radiation, λ = 0.71073 Å
Hall symbol: -P 2yn	Cell parameters from 6230 reflections
*a* = 6.5617 (7) Å	θ = 3.1–29.7°
*b* = 13.9716 (13) Å	µ = 0.08 mm^−^^1^
*c* = 12.3351 (8) Å	*T* = 298 K
β = 96.466 (7)°	Block, yellow
*V* = 1123.66 (17) Å^3^	0.42 × 0.18 × 0.15 mm
*Z* = 2	

### Data collection

**Table:**

Rigaku R-AXIS RAPID diffractometer	2684 independent reflections
Radiation source: fine-focus sealed tube	1314 reflections with *I* > 2σ(*I*)
Graphite monochromator	*R*_int_ = 0.055
ω scans	θ_max_ = 29.3°, θ_min_ = 2.9°
Absorption correction: multi-scan (*ABSCOR*; Higashi, 1995)	*h* = −7→8
*T*_min_ = 0.695, *T*_max_ = 0.856	*k* = −14→18
6268 measured reflections	*l* = −16→15

### Refinement

**Table:**

Refinement on *F*^2^	Primary atom site location: structure-invariant direct methods
Least-squares matrix: full	Secondary atom site location: difference Fourier map
*R*[*F*^2^ > 2σ(*F*^2^)] = 0.064	Hydrogen site location: inferred from neighbouring sites
*wR*(*F*^2^) = 0.146	H atoms treated by a mixture of independent and constrained refinement
*S* = 1.02	*w* = 1/[σ^2^(*F*_o_^2^) + (0.0451*P*)^2^ + 0.0549*P*] where *P* = (*F*_o_^2^ + 2*F*_c_^2^)/3
2684 reflections	(Δ/σ)_max_ < 0.001
162 parameters	Δρ_max_ = 0.34 e Å^−^^3^
0 restraints	Δρ_min_ = −0.20 e Å^−^^3^

### Special details

**Table:**

Geometry. All e.s.d.'s (except the e.s.d. in the dihedral angle between two l.s. planes) are estimated using the full covariance matrix. The cell e.s.d.'s are taken into account individually in the estimation of e.s.d.'s in distances, angles and torsion angles; correlations between e.s.d.'s in cell parameters are only used when they are defined by crystal symmetry. An approximate (isotropic) treatment of cell e.s.d.'s is used for estimating e.s.d.'s involving l.s. planes.
Refinement. Refinement of *F*^2^ against ALL reflections. The weighted *R*-factor *wR* and goodness of fit *S* are based on *F*^2^, conventional *R*-factors *R* are based on *F*, with *F* set to zero for negative *F*^2^. The threshold expression of *F*^2^ > σ(*F*^2^) is used only for calculating *R*-factors(gt) *etc*. and is not relevant to the choice of reflections for refinement. *R*-factors based on *F*^2^ are statistically about twice as large as those based on *F*, and *R*- factors based on ALL data will be even larger.

### Fractional atomic coordinates and isotropic or equivalent isotropic displacement parameters (Å^2^)

**Table:**

	*x*	*y*	*z*	*U*_iso_*/*U*_eq_	
N1	−0.2449 (3)	0.71889 (16)	0.85672 (16)	0.0593 (6)	
N2	−0.0037 (3)	0.59178 (15)	0.65051 (13)	0.0501 (6)	
N3	0.1079 (3)	0.59573 (14)	0.82913 (13)	0.0436 (5)	
C1	−0.2217 (4)	0.68146 (17)	0.75907 (18)	0.0465 (6)	
C2	−0.3657 (4)	0.6937 (2)	0.6693 (2)	0.0572 (7)	
H2	−0.3442	0.6675	0.6022	0.069*	
C3	−0.5399 (4)	0.7445 (2)	0.6803 (2)	0.0663 (8)	
H3	−0.6393	0.7525	0.6210	0.080*	
C4	−0.5664 (5)	0.7834 (2)	0.7785 (3)	0.0693 (8)	
H4	−0.6835	0.8186	0.7877	0.083*	
C5	−0.4172 (5)	0.7695 (2)	0.8636 (2)	0.0702 (9)	
H5	−0.4360	0.7968	0.9305	0.084*	
C6	−0.0367 (4)	0.62376 (17)	0.74647 (17)	0.0442 (6)	
C7	0.1763 (4)	0.53894 (18)	0.66954 (17)	0.0437 (6)	
C8	0.2838 (4)	0.48915 (19)	0.59693 (18)	0.0534 (7)	
H8	0.2371	0.4871	0.5229	0.064*	
C9	0.4599 (4)	0.4432 (2)	0.6369 (2)	0.0567 (7)	
H9	0.5342	0.4099	0.5893	0.068*	
C10	0.5305 (4)	0.4451 (2)	0.7475 (2)	0.0580 (7)	
H10	0.6509	0.4131	0.7725	0.070*	
C11	0.4249 (4)	0.49381 (19)	0.82047 (18)	0.0525 (7)	
H11	0.4713	0.4953	0.8945	0.063*	
C12	0.2482 (4)	0.54014 (17)	0.77956 (16)	0.0415 (6)	
C13	0.1227 (4)	0.61541 (18)	0.94648 (15)	0.0469 (7)	
H13B	0.0880	0.6820	0.9573	0.056*	
H13A	0.2633	0.6057	0.9782	0.056*	
C14	−0.0179 (6)	0.5522 (2)	1.0057 (2)	0.0620 (9)	
H14B	0.005 (3)	0.5697 (16)	1.0838 (18)	0.054 (7)*	
H14A	−0.173 (5)	0.544 (2)	0.978 (2)	0.099 (11)*	

### Atomic displacement parameters (Å^2^)

**Table:**

	*U*^11^	*U*^22^	*U*^33^	*U*^12^	*U*^13^	*U*^23^
N1	0.0668 (17)	0.0549 (15)	0.0575 (12)	0.0066 (13)	0.0128 (11)	−0.0001 (11)
N2	0.0581 (14)	0.0578 (14)	0.0344 (10)	−0.0035 (12)	0.0047 (9)	0.0026 (9)
N3	0.0506 (13)	0.0476 (12)	0.0328 (10)	−0.0050 (11)	0.0055 (9)	0.0015 (9)
C1	0.0516 (17)	0.0354 (14)	0.0543 (14)	−0.0035 (13)	0.0138 (12)	0.0039 (12)
C2	0.0607 (19)	0.0516 (17)	0.0591 (15)	0.0017 (15)	0.0058 (13)	0.0040 (14)
C3	0.061 (2)	0.0537 (18)	0.0818 (19)	0.0009 (16)	−0.0008 (16)	0.0112 (16)
C4	0.0522 (19)	0.0499 (18)	0.108 (2)	0.0101 (15)	0.0175 (17)	0.0112 (18)
C5	0.085 (2)	0.056 (2)	0.0748 (19)	0.0079 (18)	0.0293 (18)	−0.0059 (15)
C6	0.0505 (16)	0.0444 (15)	0.0381 (12)	−0.0069 (13)	0.0066 (11)	0.0047 (11)
C7	0.0423 (15)	0.0469 (15)	0.0423 (13)	−0.0043 (12)	0.0069 (11)	0.0043 (11)
C8	0.0600 (18)	0.0629 (19)	0.0387 (12)	−0.0069 (16)	0.0115 (12)	−0.0005 (12)
C9	0.0580 (19)	0.0595 (18)	0.0566 (15)	0.0009 (15)	0.0234 (13)	−0.0076 (14)
C10	0.0474 (17)	0.0620 (18)	0.0649 (16)	0.0016 (14)	0.0077 (13)	0.0059 (14)
C11	0.0534 (17)	0.0628 (18)	0.0399 (12)	−0.0063 (15)	−0.0005 (11)	0.0032 (13)
C12	0.0472 (15)	0.0411 (14)	0.0381 (12)	−0.0051 (13)	0.0129 (11)	0.0016 (11)
C13	0.0600 (16)	0.0503 (16)	0.0303 (11)	−0.0095 (13)	0.0046 (10)	−0.0044 (11)
C14	0.095 (3)	0.0579 (17)	0.0345 (13)	−0.0119 (19)	0.0139 (14)	−0.0042 (14)

### Geometric parameters (Å, º)

**Table:**

N1—C1	1.337 (3)	C7—C12	1.386 (3)
N1—C5	1.344 (3)	C7—C8	1.388 (3)
N2—C6	1.306 (3)	C8—C9	1.364 (3)
N2—C7	1.391 (3)	C8—H8	0.9300
N3—C6	1.370 (3)	C9—C10	1.390 (3)
N3—C12	1.397 (3)	C9—H9	0.9300
N3—C13	1.466 (2)	C10—C11	1.376 (3)
C1—C2	1.383 (3)	C10—H10	0.9300
C1—C6	1.480 (3)	C11—C12	1.373 (3)
C2—C3	1.365 (4)	C11—H11	0.9300
C2—H2	0.9300	C13—C14	1.523 (3)
C3—C4	1.357 (4)	C13—H13B	0.9700
C3—H3	0.9300	C13—H13A	0.9700
C4—C5	1.366 (4)	C14—C14^i^	1.486 (6)
C4—H4	0.9300	C14—H14B	0.99 (2)
C5—H5	0.9300	C14—H14A	1.04 (3)
			
C1—N1—C5	116.4 (2)	C9—C8—H8	120.9
C6—N2—C7	104.66 (19)	C7—C8—H8	120.9
C6—N3—C12	105.58 (17)	C8—C9—C10	121.4 (2)
C6—N3—C13	130.1 (2)	C8—C9—H9	119.3
C12—N3—C13	124.27 (19)	C10—C9—H9	119.3
N1—C1—C2	122.4 (2)	C11—C10—C9	121.0 (3)
N1—C1—C6	119.0 (2)	C11—C10—H10	119.5
C2—C1—C6	118.5 (2)	C9—C10—H10	119.5
C3—C2—C1	119.3 (3)	C12—C11—C10	117.3 (2)
C3—C2—H2	120.4	C12—C11—H11	121.3
C1—C2—H2	120.4	C10—C11—H11	121.3
C4—C3—C2	119.3 (3)	C11—C12—C7	122.3 (2)
C4—C3—H3	120.3	C11—C12—N3	132.2 (2)
C2—C3—H3	120.3	C7—C12—N3	105.5 (2)
C3—C4—C5	118.4 (3)	N3—C13—C14	112.9 (2)
C3—C4—H4	120.8	N3—C13—H13B	109.0
C5—C4—H4	120.8	C14—C13—H13B	109.0
N1—C5—C4	124.2 (3)	N3—C13—H13A	109.0
N1—C5—H5	117.9	C14—C13—H13A	109.0
C4—C5—H5	117.9	H13B—C13—H13A	107.8
N2—C6—N3	113.9 (2)	C14^i^—C14—C13	114.4 (3)
N2—C6—C1	120.2 (2)	C14^i^—C14—H14B	109.1 (14)
N3—C6—C1	125.8 (2)	C13—C14—H14B	106.8 (13)
C12—C7—C8	119.8 (2)	C14^i^—C14—H14A	90.9 (18)
C12—C7—N2	110.35 (19)	C13—C14—H14A	122.1 (16)
C8—C7—N2	129.9 (2)	H14B—C14—H14A	113 (2)
C9—C8—C7	118.3 (2)		

Symmetry code: (i) −*x*, −*y*+1, −*z*+2.
